# Editorial: Mass spectrometry-based proteogenomics – advances in mutant proteomics and clinical oncology

**DOI:** 10.3389/fonc.2024.1383838

**Published:** 2024-03-21

**Authors:** Akos Végvári, Toshihide Nishimura

**Affiliations:** ^1^ Department of Medical Biochemistry and Biophysics, Karolinska Institutet (KI), Stockholm, Sweden; ^2^ Department Chest Surgery, St. Marianna University School of Medicine, Kawasaki, Japan

**Keywords:** mass spectrometry-based proteogenomics, mutant proteomics, clinical oncology, biomarker, treatment-strategy development

Advances in mass spectrometry (MS) based proteomics have been instrumental in emerging areas where proteome alterations need to be described in detail. While technological progress in mass spectrometers has been paramount for accurate data acquisition, there is still a need for development in data interpretation. The emergence of proteogenomics has helped narrow the gap between protein structure and function by identifying proteoforms and their relationship to molecular mechanisms, particularly in disease progression.

The greatest challenge of proteogenomics in clinical oncology is to understand the systems biology of tumor subtypes by integrating proteomics data with genomics and transcriptomics data, which can ultimately lead to the identification of better clinical biomarkers in tumor development, novel treatment strategies, drug discovery, and improved treatment response. Cancer diseases are often characterized by high mutation rates, which are closely related to the physiological and pathological characteristics of individual patients. A variety of driver mutations have been discovered, which has in turn led to the development of targeted treatment strategies. These efforts have been aided by the use of transcriptome data, with thousands of new or unassessed splice junctions, sequence variants, chimeric transcripts, non-coding RNAs, gene fusions, and RNA editing events.

Proteomics data is mostly limited to the identification and quantification of consensus sequences and may aim to describe post-translational modifications known to be related to specific protein functions. In contrast, mutant proteoforms are often less investigated. Clinical proteomics studies are indispensable for monitoring genomic changes that reflect the functional interaction of protein-protein networks. This can be enabled by MS-based proteogenomics, which detects and quantifies novel peptides expressed in specific samples and mutant proteins. The number of nsSNPs is estimated to be >3 million, suggesting that single amino acid variants (SAAVs) are widely distributed in the human proteome (1).

The majority of human cancers are characterized by high mutation rates, with numerous mutant proteins being expressed exclusively in cancer cells and being closely associated with the physiological and pathological traits of individuals. Furthermore, the allele-specific gene expressions in the heterozygous state are also associated with various traits in individuals (2, 3). An orthogonal partial least squares discriminant analysis (OPLS-DA) of mutant proteins expressed in lung adenocarcinoma patients with representative EGFR driver mutations (Ex19del, L858R, and no Ex19del/L858R) demonstrated the profound differences in distance between these EGFR mutation groups (4). Such proteomics data can be obtained on a high-performance MS equipped with data-independent acquisition (DIA) and an automated software suite (DIA-NN), which enables fast and reliable protein identification (5). High-throughput label-free ion intensity-based quantitative proteomics, and can discover key disease-related proteins and therapeutic targets in oncology.

In this Research Topic, we are pleased to present contributions on MS-based proteogenomics and its development in clinical oncology, which may improve our understanding of tumor biology and has important implications for patient care.

Wang et al. performed serum proteomic profiling on patients with prostate cancer (PCa) and benign prostatic hyperplasia (BPH) using DIA-MS to discover putative biomarkers for early-stage PCa and also to discriminate between aggressive and non-aggressive disease. The authors demonstrated that prostate-specific antigen (PSA) plus the combination of osteopontin (secreted phosphoprotein 1, SPP1) and ceruloplasmin (CP) could effectively distinguish between diseases with high and low Gleason scores compared with diagnosis by PSA alone, and serum SPP1 and CP could differentiate aggressive PCa (especially with high Gleason scores) from non-aggressive disease.

Rappu et al. analyzed cancer proteomics datasets in public databases (PRIDE, MassIVE, and CPTAC) for citrullinated matrisome proteins, and structural proteins in the extracellular matrix (ECM), and refined high-performance MS for identifications in three-dimensional cell cocultures of fibroblasts and numerous cancer cell lines. Their systematic study revealed that citrullination of matrisome proteins is related to inflammation and their function may be impaired in cancer because arginine residues are often located at the critical protein-protein interaction sites.

Plasma cell dyscrasias (PCD) encompass a broad spectrum of diseases ranging from asymptomatic monoclonal gammopathy of undetermined significance to life-threatening diseases. Detection of M-protein has been a current practice in the diagnosis and post-treatment monitoring of PCD which is characteristically accompanied by the clonal expansion of plasma cells, secreting monoclonal immunoglobin components (M-protein). Li et al. developed a novel immuno-enrichment-free MS-based method for the detection and isotypic identification of M-proteins and type confirmation, taking advantage of MALDI-TOF-MS, which is capable of detecting high molecular weight biomolecules in the mass range of 5,000–30,000 *m/z*, which demonstrated excellent analytical performance and throughput.

Chen et al. employed an ultrahigh-performance liquid chromatography-tandem high-resolution MS (UHPLC-HRMS/MS) method to comprehensively measure lipid species in human serum samples from patients with colorectal adenoma (CA) and carcinoma (CRC). The authors demonstrated significant differences in serum lipid profiles between these groups, discovering 85 differential lipid species (*p*-value < 0.05 and fold change > 1.50 or < 0.67) were discovered. Thus, they first undertook a lipidomics profile using serum intended to identify screening lipid biomarkers to discriminate between CA and CRC, from which seven lipid species were proposed as potential biomarkers (with the area under the curve (AUC) > 0.800). Their results suggest that dysregulated lipid metabolism of fatty acids (FAs), phosphatidylcholines (PCs), and triacylglycerols (TAGs) should be closely connected with the malignant transformation process from CA to CRC.

Solid-predominant lung adenocarcinoma (SPA) is a high-risk subtype with a poor prognosis and an unsatisfactory response to chemotherapy and targeted therapy for lung adenocarcinoma. Nishimura et al. conducted an MS-based proteomic analysis of cancerous cells laser-microdissected from formalin-fixed paraffin-embedded tissues to identify disease-related co-expression networks associated with the SPA subtype, followed by a weighted network correlation analysis (WGCNA) and their upstream regulator analysis. Their proteome data and upstream analysis suggested the involvement of highly activated oncogenic regulators, the redox master regulator NFE2L2, adaptive immune response regulators, and highly inhibited leukocyte immunoglobulin-like receptor subfamily B member 2 (LILRB2), whose counterpart, the immune checkpoint molecule HLA-G, was highly expressed in the SPA subtype. Together, the authors suggested that SPA may be more amenable to immunotherapy, but less amenable to chemo- and targeted therapy, which could improve treatment and benefit patients with solid predominant lung adenocarcinoma.

## Author contributions

AV: Writing – review & editing, Writing – original draft, Conceptualization. TN: Writing – review & editing, Writing – original draft, Conceptualization.
